# Mandibular CT-Based radiomorphometric and density indices for opportunistic osteoporosis screening

**DOI:** 10.1186/s12903-026-07829-2

**Published:** 2026-02-06

**Authors:** Yakup Şen, Sümeyye Coşgun Baybars, Seda Soğukpınar Karaağaç

**Affiliations:** 1 Department of Oral and Maxillofacial Radiology, Faculty of Dentistry, Fırat University, Elazığ, Turkey; 2 Department of Radiology, Faculty of Medicine, Fırat University, Elazığ, Turkey

**Keywords:** Osteoporosis, DEXA, Bone mineral density, Computed tomography, Mandibular index, Hounsfield unit

## Abstract

**Purpose:**

This study aimed to evaluate the association between mandibular radiomorphometric indices (CTMI, CTI(S), CTI(I)) and trabecular Hounsfield Unit (HU) values obtained from computed tomography (CT) scans with osteoporosis as defined by dual-energy X-ray absorptiometry (DEXA).

**Methods:**

This retrospective study was conducted at the Department of Radiology, Faculty of Medicine, Fırat University, using routine head and neck CT scans. All participants underwent DEXA at the L1–L4 vertebral levels according to World Health Organization (WHO) criteria. The osteoporosis group consisted of 50 patients with a T-score ≤ −2.5 and a clinical diagnosis of osteoporosis by the Physical Therapy Department, while the control group comprised 50 individuals with T-scores ≥ −1 and no diagnosis of osteoporosis. Bilateral measurements of CTMI, CTI(S), and CTI(I) were averaged, and HU values were recorded from predefined mandibular regions.

**Results:**

All radiomorphometric indices and HU values were significantly lower in osteoporotic individuals compared to the control group. ROC analysis demonstrated threshold values for CTMI ≤ 3.35 mm (sensitivity: 86%, specificity: 98%), CTI(I) ≤ 0.36 (sensitivity: 78%, specificity: 92%), and CTI(S) ≤ 0.27 (sensitivity: 78%, specificity: 98%). Threshold HU values were observed as ≤ 337 for the mandibular angle, ≤ 209 for the mandibular corpus, and ≤ 470 for the interradicular area between teeth 3 and 4. Among these regions, the mandibular angle showed the highest area under the curve (AUC: 0.857; sensitivity: 90%).

**Conclusion:**

Mandibular CT-derived radiomorphometric and densitometric measurements are associated with osteoporosis as defined by DEXA and may provide complementary information within an opportunistic screening framework.

**Clinical trial registration:**

Not applicable. This retrospective study did not require registration in a clinical trial registry.

## Introduction

Osteoporosis is a chronic and progressive skeletal disorder characterized by reduced bone mineral density (BMD), leading to increased bone porosity and compromised structural integrity of the skeleton [1]. According to the World Health Organization (WHO), as of 2019, an estimated 25.5 million women and 6.5 million men in the European Union, the United Kingdom, and Switzerland were diagnosed with osteoporosis [2]. In Turkey, the FRACTURK study reported a prevalence of osteoporosis of 24.8% and osteopenia of 49.6% among individuals over 50 years of age [3]. Research indicates that the annual loss of BMD ranges between 0.86% and 1.12% in women, and between 0.04% and 0.9% in men [4].

When diagnosed at an early stage, osteoporosis may be partially preventable or its progression can be significantly delayed prior to substantial bone loss [5]. With appropriate screening and preventive strategies, proximal femur fractures can be reduced by 20%, vertebral compression fractures by 12%, and peripheral fractures by 25% [4, 6]. Currently, the most widely used and gold-standard method for diagnosing osteoporosis is Dual-Energy X-ray Absorptiometry (DEXA) [7]. However, the limited accessibility and high cost of DEXA devices present challenges to early diagnosis, particularly in developing countries [8].

The systemic effects of osteoporosis may also impact the jawbones. Bone loss often begins in the alveolar bone and can be detected through the evaluation of mandibular structures. Several studies have demonstrated a significant correlation between mandibular bone mass and total body skeletal mass [4, 9]. Although panoramic radiographs are frequently utilized for this purpose, their diagnostic sensitivity is limited due to two-dimensional constraints such as superimposition and distortion. In contrast, computed tomography (CT) provides high-resolution imaging of both cortical and trabecular bone structures and allows for quantitative analysis through Hounsfield Unit (HU) measurements. Nonetheless, CT-based studies focusing on mandibular bone changes in osteoporosis remain relatively scarce in the literature.

The aim of this study was to evaluate the association between mandibular CT-derived radiomorphometric indices (CTMI, CTI(S), CTI(I)) and trabecular HU values with osteoporosis as defined by DEXA, by comparing patients with osteoporosis and healthy controls. In addition, the potential contribution of mandibular CT imaging as a complementary and opportunistic screening tool for the early identification of individuals at risk for osteoporosis was explored.

## Materials and methods

This retrospective study was conducted between January 2020 and December 2024 at the Department of Radiology, Faculty of Medicine, Fırat University, using routine head and neck CT scans. All CT examinations included in this study were obtained for routine diagnostic purposes unrelated to osteoporosis evaluation, such as maxillofacial or head-and-neck assessment. No CT scan was performed specifically for osteoporosis screening. The use of mandibular CT data in this study therefore represents an opportunistic assessment based on images acquired for other clinical indications. All participants were of Turkish ethnicity and had undergone DEXA of the L1–L4 vertebrae according to the criteria set by the WHO. The osteoporosis group consisted of 50 patients with T-scores ≤ − 2.5 and a confirmed clinical diagnosis of osteoporosis by the Department of Physical Therapy. The control group comprised 50 individuals with T-scores ≥ − 1, considered systemically healthy with respect to osteoporosis. Subjects with osteopenia (− 2.5 < T-score < − 1) were excluded to maintain a clear distinction between osteoporosis and normal bone density groups and to reduce potential overlap in CT-based measurements.

Based on power analysis, a minimum of 48 participants per group was determined to be sufficient to achieve 80% power at a 5% significance level. The study protocol was approved by the Non-Interventional Research Ethics Committee of Fırat University (Date: 27.12.2024; Decision No: 29955).

CT scans were obtained using a General Electric Revolution scanner with parameters set at 120 kVp and 80 mAs, with an average scanning duration of 10.62 s. Images were reconstructed with a slice thickness of 1.0 mm using a bone reconstruction algorithm. DEXA measurements were performed using the Lunar Prodigy device (GE Medical Systems), and analyses were carried out using Encore v13.0 software. The interval between CT and DEXA scans did not exceed 6 months, minimizing the impact of potential metabolic variations in bone density.

Inclusion criteria encompassed individuals over 50 years of age with no known metabolic, endocrine, or connective tissue disorders, and no hepatic or renal failure that could influence bone metabolism. Individuals taking medications known to affect bone metabolism—such as corticosteroids, anticonvulsants, heparin, thyroid hormones, diuretics, or methotrexate—were excluded. Written informed consent was obtained from all participants.

CT scans were first assessed for image quality. Subjects with prior mandibular resection or those with cystic or tumoral lesions were excluded. Measurements were performed on CT scans with an adequate field of view (FOV) covering both sides of the mandible, including the inferior cortex, mental foramen, and extending to the ramus. Anatomical structures such as lamina dura, teeth, periodontal ligament, and the mandibular canal were excluded from the measurement regions; only healthy trabecular bone was analyzed.

### Digital radiomorphometric measurements

**Fig. 1:**
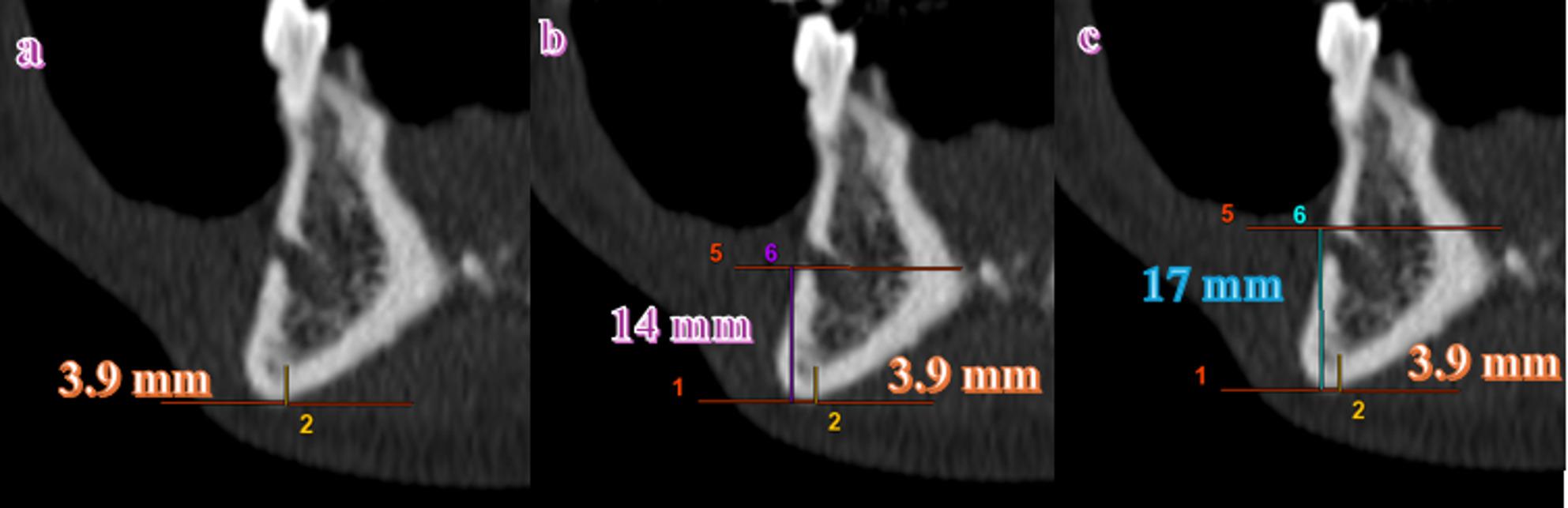
CT image of a patient from the study group demonstrating (on coronal slices): (**a**) CTMI measurement: 3.9 mm, (**b**) CTI(I) calculation: 3.9 / 14 = 0.278, (**c**) CTI(S) calculation: 3.9 / 17 = 0.229. (Numerical labels visible within the ROI (Region of Interest) are software-generated display markers and have no analytical significance)

- CTMI (Computed Tomography Mental Index): Represents the cortical thickness measured in the coronal plane at the level of the mental foramen, between tangents drawn along the superior and inferior cortical borders. The final value was calculated as the average of bilateral measurements (Fig. 1a).
- CTI (Computed Tomography Mandibular Index):
  - CTI(S): Ratio of cortical thickness to the vertical distance between the inferior mandibular border and the superior border of the mental foramen (Fig. 1c).
  - CTI(I): Ratio of cortical thickness to the vertical distance between the inferior mandibular border and the inferior border of the mental foramen (Fig. 1b).Both indices were calculated as the mean of bilateral measurements.

### Densitometric measurements

Trabecular bone density measurements were performed exclusively on sagittal CT slices obtained at predefined and standardized anatomical levels of the mandible. Circular ROIs were placed in anatomically appropriate, homogeneous, and measurement-safe trabecular bone areas, while carefully avoiding cortical bone, dental roots, the mandibular canal, and overlapping anatomical structures.

The ROIs were placed with diameters of approximately 2–3 mm, with particular attention to anatomical safety to prevent inclusion of adjacent structures.

Due to regional morphological differences in trabecular bone across the mandible, ROI sizes were not identical at all measurement sites. Instead, ROI sizes were adjusted within a narrow and predefined range, based on anatomical and methodological criteria, to ensure reliable density measurements while maintaining anatomical safety. The same ROI placement criteria and standardized anatomical reference points were consistently applied across all subjects to ensure methodological consistency.

**Fig. 2:**
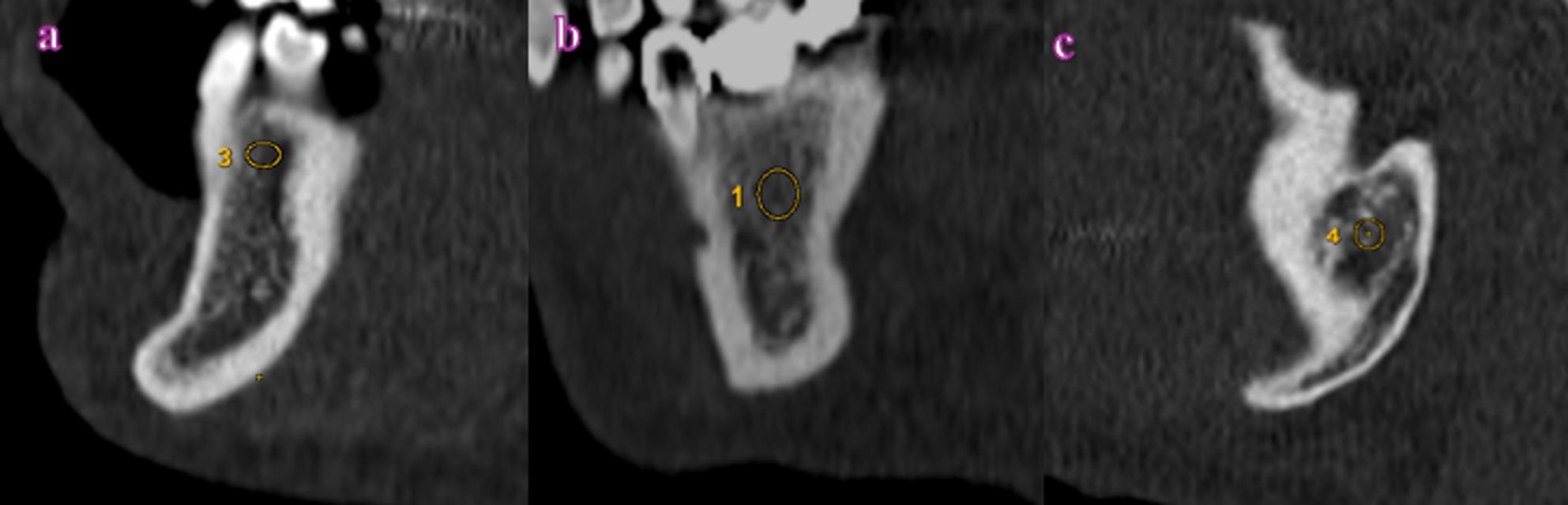
Densitometric measurements from CT images (on sagittal slices): (**a**) Interdental region between teeth 3 and 4 (214 HU), (**b**) Area inferior to the apex of tooth #6 (317 HU), (**c**) Mandibular angle region (405 HU). (Numerical labels visible within the ROIs are software-generated display markers and have no analytical significance)

- Interdental area between teeth 3 and 4 (Fig. 2a),
- Mandibular corpus (2 mm inferior to the apex of tooth #6) (Fig. 2b),
- Mandibular angle region (angulus) (Fig. 2c).

### Statistical analysis

All statistical analyses were performed using IBM SPSS Statistics version 22. The normality of data distribution was assessed using the Kolmogorov-Smirnov and Shapiro-Wilk tests. Descriptive statistics (minimum, maximum, mean, standard deviation, median, and frequency) were calculated. For quantitative variables, comparisons between two groups were made using the Student’s t-test for normally distributed data, and the Mann-Whitney U test for non-normally distributed data. For categorical variables, the Continuity Correction (Yates’ correction) was applied.

Optimal cut-off values for diagnostic parameters were determined using receiver operating characteristic (ROC) curve analysis. Pearson correlation analysis was used to assess relationships between normally distributed variables. A *p*-value of < 0.05 was considered statistically significant.

## Results

A total of 100 participants (23 males, 77 females), aged 50–98 years (mean ± SD: 61.16 ± 9.64 years), were included in the study. The osteoporosis group comprised 10 males (20%) and 40 females (80%), whereas the control group included 13 males (26%) and 37 females (74%). The mean age was significantly higher in the osteoporosis group than in the control group (65.90 ± 10.17 vs. 56.42 ± 6.21 years, *p* = 0.001), while gender distribution did not differ significantly between groups (*p* = 0.635).

**Table 1:** Evaluation of CTI(S), CTI(I), and CTMI between groups

	Patient	Control	
	Mean ± SD	Mean ± SD	*P*
CTI(S)	0.226 ± 0.08	0.387 ± 0.07	0.001*
CTI(I)	0.295 ± 0.12	0.486 ± 0.09	0.001*
CTMI	2.713 ± 0.77	4.839 ± 0.85	0.001*

Student’s t-test

*p < 0.05

Radiomorphometric indices were significantly reduced in the osteoporosis group compared with controls (Table 1):

- CTI(S): 0.226 ± 0.08 vs. 0.387 ± 0.07 (*p* = 0.001)
- CTI(I): 0.295 ± 0.12 vs. 0.486 ± 0.09 (*p* = 0.001)
- CTMI: 2.713 ± 0.77 mm vs. 4.839 ± 0.85 mm (*p* = 0.001)

**Table 2:** ROC analysis results for indices in osteoporosis diagnosis

	AUC	SE	95% CI	*P*	Cut-off Point	Sensitivity	Specificity
CTI(S)	0.922	0.03	0.851–0.966	0.001*	≤ 0.27	78.0	98.0
CTI(I)	0.886	0.03	0.807–0.941	0.001*	≤ 0.36	78.9	92.0
CTMI	0.963	0.02	0.905–0.991	0.001*	≤ 3.35	86.0	98.0

ROC curve analysis demonstrated high diagnostic accuracy for these indices (Table 2):

- CTI(S): AUC = 0.922 (SE = 0.03; 95% CI: 0.851–0.966; *p* = 0.001); cut-off ≤ 0.27; sensitivity 78%, specificity 98%
- CTI(I): AUC = 0.886 (SE = 0.03; 95% CI: 0.807–0.941; *p* = 0.001); cut-off ≤ 0.36; sensitivity 78%, specificity 92%
- CTMI: AUC = 0.963 (SE = 0.02; 95% CI: 0.905–0.991; *p* = 0.001); cut-off ≤ 3.35 mm; sensitivity 86%, specificity 98%

**Fig. 3:**
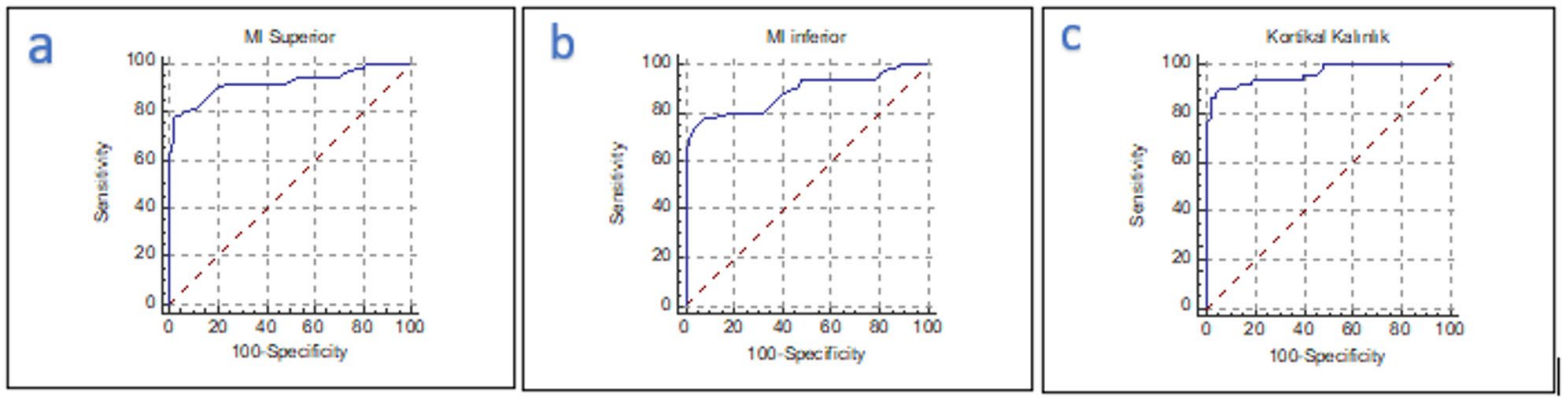
ROC curve analysis was performed for (**a**) CTI(S), (**b**) CTI(I), and (**c**) CTMI

As illustrated in Fig. 3, ROC curve analysis demonstrated high diagnostic accuracy for CTI(S), CTI(I), and CTMI.

**Table 3:** Comparison of density measurements between the groups

Density	Patient	Control	
	Mean ± SD (median) (Min-Max)	Mean ± SD (median) (Min-Max)	*P*
Angle regions	199.28 ± 124.24 (177.5)	451.28 ± 200.18 (429)	0.001*
	(30–566)	(148–886)	
Inferior to the first molar	196.0 ± 166.49 (146)	289.5 ± 161.18 (274)	0.001*
	(30–914)	(57–857)	
3–4 between intramedullary area	389.80 ± 252.51 (328.5)	544.04 ± 228.86 (503)	0.001*
	(99-1662)	(146–958)	

Mann Whitney U test

*p < 0.05

Mandibular trabecular bone density was also significantly lower in the osteoporosis group (Table 3):

- Angle region: 199.28 ± 124.24 HU vs. 451.28 ± 200.18 HU (*p* = 0.001)
- Inferior to the first molar: 196.00 ± 166.49 HU vs. 289.50 ± 161.18 HU (*p* = 0.001)
- In terradicular area (teeth 3–4): 389.80 ± 252.51 HU vs. 544.04 ± 228.86 HU (*p* = 0.001)

**Table 4:** ROC analysis results of bone density measurements for the diagnosis of osteoporosis

	AUC	SE	95% CI	*P*	Cut-off Point	Sensitivity	Specificity
Angle region	0.857	0.04	0.773–0.919	0.001*	≤ 337	90	70
Inferior to the first molar	0.710	0.05	0.611–0.797	0.001*	≤ 209	68	68
3–4 between intramedullary area	0.718	0.05	0.619–0.803	0.001*	≤ 470	80	58

ROC analysis for HU measurements yielded the following diagnostic performance (Table 4):

- Angle region: AUC = 0.857 (SE = 0.04; 95% CI: 0.773–0.919; *p* = 0.001); cut-off ≤ 337 HU; sensitivity 90%, specificity 70%
- Inferior to the first molar: AUC = 0.710 (SE = 0.05; 95% CI: 0.611–0.797; *p* = 0.001); cut-off ≤ 209 HU; sensitivity 68%, specificity 68%
- Interradicular area: AUC = 0.718 (SE = 0.05; 95% CI: 0.619–0.803; *p* = 0.001); cut-off ≤ 470 HU; sensitivity 80%, specificity 58%

**Fig. 4:**
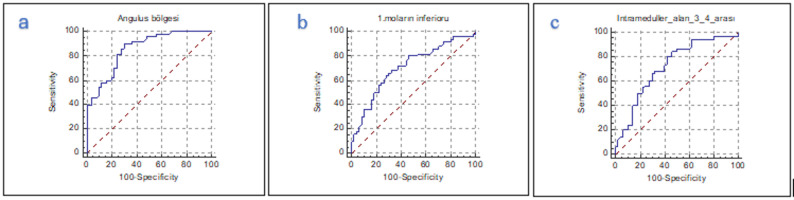
ROC curve analysis of HU values for (**a**) The angulus region, (**b**) Inferior of the first molar (**c**) The region between teeth 3 and 4

As illustrated in Fig. 4, ROC curve analysis of HU values in the angulus region, inferior to the first molar, and interradicular area demonstrated their diagnostic utility in osteoporosis assessment.

## Discussion

### Mandibular CT-based findings in osteoporosis

In this study, mandibular bone changes in patients diagnosed with osteoporosis were evaluated using CT-derived radiomorphometric indices [CTMI, CTI(S), CTI(I)] and HU values. The aim was to investigate whether mandibular CT findings could serve as a complementary assessment tool within an opportunistic screening framework for osteoporosis.

### Limitations of DEXA and the potential of dental imaging

DEXA has long been regarded as the gold standard for osteoporosis diagnosis, owing to its high accuracy and reproducibility [10, 11]. However, the limited accessibility and high cost of DEXA scanners, especially in developing countries, can reduce screening frequency and delay diagnosis. In this context, CT scans obtained for routine head and neck imaging may offer valuable insights into systemic bone health [12].

### Radiomorphometric findings compared with the literature

In the present study, CTMI, CTI(S), and CTI(I) values were found to be significantly lower in the osteoporosis group. Similar reductions have been reported by Koh and Kim in a cone-beam computed tomography (CBCT)-based study conducted on postmenopausal women, in which the CTMI value was 2.23 mm in the osteoporosis group and 3.22 mm in the control group [13]. However, in our study, the CTMI value was measured as 2.71 mm in the osteoporosis group and 4.83 mm in the control group.

Geibel et al. established diagnostic threshold values for CBCT-derived mandibular indices in DEXA-confirmed osteoporosis cases, reporting values of < 3.0 mm for CTMI, < 0.18 for CTI(S), and < 0.23 for CTI(I) [14]. In the present study, higher threshold values were identified (CTMI ≤ 3.35 mm, CTI(S) ≤ 0.27, CTI(I) ≤ 0.36), which yielded greater sensitivity and specificity. These findings support the feasibility of using these indices as effective screening tools for osteoporosis. The observed differences in threshold values may be attributed to the larger sample size of our study, a more balanced gender distribution, the use of higher-resolution CT equipment, and the implementation of stricter exclusion criteria.

While conventional CT allows for quantitative and reproducible assessment of cortical and trabecular bone structures, CBCT, despite offering lower radiation exposure and high spatial resolution, presents technical limitations in the absolute evaluation of bone density. Although CBCT-based radiomorphometric indices have been reported to be useful for osteoporosis screening, these assessments are largely qualitative or semi-quantitative in nature. Therefore, CT-based radiomorphometric indices provide a more quantitative and reproducible approach, particularly in the context of opportunistic osteoporosis assessment [15, 16].

### Advantages of CT compared with panoramic radiography

Numerous studies have demonstrated that panoramic radiographs may be useful in the early detection of osteoporosis [17–20]. However, being a two-dimensional imaging modality, panoramic radiography is subject to several technical limitations such as superimposition, magnification, distortion, and variability in repeated measurements. These issues can negatively impact the accuracy and reliability of the assessments. Moreover, the inability to evaluate buccolingual dimensions may lead to missed or misinterpreted cortical bone loss [21, 22].

In contrast, the CT modality used in our study offers several important advantages, including high image resolution, true-to-scale sectional measurements, and the ability to assess cortical and trabecular bone separately. Additionally, CT allows for quantitative densitometric analysis using HU. The three-dimensional capabilities of CT enable a more objective, reproducible, and anatomically precise evaluation of mandibular bone structures compared to panoramic radiography.

### Objectivity and potential utility of HU measurements

HU values are numerical indicators of bone density derived directly from CT imaging. Unlike HU values in CBCT systems, which lack standardization and are therefore unreliable [15, 16], conventional CT provides reproducible and objective measurements. Previous studies have demonstrated significant correlations between HU values and systemic osteoporosis, particularly in the lumbar vertebrae [23–25] and proximal humerus [26].

Our study is among the few that have explored this correlation in detail within the mandibular region. HU values obtained from the mandibular angle, corpus, and the interradicular area between teeth 3 and 4 were significantly lower in the osteoporosis group. Among these regions, the mandibular angle showed the highest discriminatory performance with an AUC of 0.857 and a sensitivity of 90%. This may be attributed to the anatomical and functional role of the angle region, which is subjected to masticatory forces and may therefore be more sensitive to systemic skeletal changes [27, 28].

### Consistency of our findings with the literature

In the study by Kim et al., CT-based measurements of 331 lumbar vertebrae from 120 patients demonstrated that HU values had a sensitivity of 82.5% for diagnosing osteoporosis, with a proposed diagnostic threshold of 95 HU [24]. Similarly, Pickhardt et al. compared HU values derived from CT with DEXA results in a large cohort of 1867 patients. They found that a threshold of 160 HU at the L1 vertebra provided 90% sensitivity for distinguishing osteoporosis from osteopenia, while a threshold of 110 HU achieved 90% specificity [25].

In our study, HU measurements from three different mandibular sites angle, corpus, and the interradicular region between teeth 3 and 4 yielded threshold values of ≤ 337, ≤209, and ≤ 470, respectively. The corresponding sensitivity and specificity values were 90%–70% for the angle region, 68%–68% for the corpus, and 80%–58% for the interradicular area. These findings suggest that mandibular CT measurements, particularly those from the angle region, may have significant potential utility for osteoporosis screening.

In a study by Joseph et al., 25 patients underwent both lumbar CT and DEXA within a 6-month period and were classified as normal, osteopenic, or osteoporotic. The average HU values were 133.0 for the normal group, 100.8 for the osteopenic group, and 78.5 for the osteoporotic group. Strong correlations between HU values and both BMD and T-scores were observed [23]. In our study, mean HU values in the osteoporosis group were 199 (angle), 196 (corpus), and 389 (interradicular region), all significantly lower than those in the control group (451, 289, and 544, respectively). Differences in mean and threshold values between our study and those by Joseph, Kim, and Pickhardt may be attributed to differences in anatomical measurement sites, age distribution, sample size, and scanner calibration protocols.

In a CT-based study by Cheade et al., positive correlations were observed between BMD values from cervical vertebrae and those from the maxillary and mandibular regions, consistent with our findings [9]. Likewise, Lee et al. found statistically significant positive correlations between mandibular cortical HU values and systemic BMD, and they reported significantly reduced HU values in osteoporotic individuals, in line with our results [29].

In another study by Chai et al., HU values were measured from both right and left mandibular canine regions in 122 sites, and concurrent DEXA scans of the L1–L4 vertebrae were obtained. Their findings indicated that HU values from the mandibular region were predictive of osteoporosis as defined by spinal T-scores, and they proposed an optimal threshold of approximately 460 HU for this region [30]. In agreement with these findings, our study also observed a significant relationship between spinal T-scores and HU values in the mandibular canine-premolar region, where a threshold of 470 HU yielded 80% sensitivity in differentiating osteoporotic individuals within a screening context.

### Clinical implications and contributions to dentistry

CT examinations routinely acquired for various diagnostic purposes can provide quantitative information on mandibular bone structure without requiring additional imaging or incurring extra costs. Such data may be considered within an opportunistic screening framework to aid in the identification of individuals who may be at increased risk for osteoporosis [31–33]. However, it should be emphasized that this approach is not intended to serve as a diagnostic method, but rather as a preliminary assessment tool aimed at increasing clinical awareness.

In contemporary dental practice, CT imaging is widely used for diverse clinical indications. In this context, particularly in elderly patients, measurements of mandibular bone density may offer supportive information regarding changes potentially associated with osteoporosis as defined by DEXA. Nevertheless, mandibular CT-based measurements are not intended to replace DEXA; instead, they should be regarded as a complementary approach that may provide additional clinical insight into osteoporosis risk based on already available imaging data.

### Strengths and novel contributions

The principal strengths of the present study include:

- The use of bilateral and quantitative CT-based measurements of the mandible,
- Systematic analysis of three distinct mandibular anatomical regions,
- Retrospective evaluation of existing CT data without exposing patients to additional radiation,
- A methodological focus on examining the association between mandibular CT indices and osteoporosis as identified by DEXA.

Furthermore, compared with similar studies in the literature, the relatively larger sample size enhances the statistical power of the analysis and supports the robustness and reliability of the findings.

### Limitations and future research

This study has several limitations. First, all measurements were performed by a single observer, and inter-observer variability could not be assessed. In addition, factors that may influence bone density such as dental status, including edentulism and prosthesis use were not incorporated into the analysis.

Age represents a major biological determinant of bone remodeling and mineral density loss, affecting both cortical and trabecular bone compartments. Previous studies have shown that mandibular cortical thickness and trabecular bone density progressively decrease with advancing age, independent of systemic osteoporosis status [34, 35]. In the present study, the osteoporosis group exhibited a significantly higher mean age than the control group, which may have contributed to the observed reductions in mandibular cortical indices and trabecular HU values. Accordingly, age should be considered a potential confounding factor when interpreting CT-based measurements, and the findings should be evaluated within this context.

Although CT provides standardized HU measurements, absolute HU-based threshold values may vary depending on scanner type, acquisition parameters, reconstruction algorithms, FOV, and ROI size. In the absence of phantom-based calibration, HU values should therefore be interpreted within the context of the specific imaging protocol used. In the present study, HU measurements were primarily employed for comparative analysis between groups rather than for defining universal diagnostic cut-off values. These results support the potential utility of mandibular HU measurements as part of an opportunistic screening approach, while underscoring the need for protocol standardization and calibration in future investigations.

Although osteopenia is clinically relevant in screening settings, it was deliberately excluded from the present study to preserve diagnostic contrast between clearly defined osteoporosis and normal bone density groups.

Future studies should include multiple independent observers with formal intra- and inter-rater reliability analyses, longitudinal follow-up designs to better evaluate age-related changes in mandibular bone structure, and standardized CT acquisition protocols including scanner calibration and phantom-based HU normalization. In addition, incorporating dental status parameters and larger, multi-center cohorts would further enhance the methodological robustness and clinical interpretability of mandibular CT-based osteoporosis assessment.

## Conclusion

The findings of this study indicate that mandibular radiomorphometric and densitometric measurements derived from CT are associated with osteoporosis as defined by DEXA and reflect structural changes in mandibular bone tissue. CT-based measurements obtained from routine head and neck imaging may provide supplementary information within an opportunistic screening framework and help identify individuals who may benefit from further dedicated osteoporosis evaluation. Importantly, these measurements are not intended to replace DEXA, but rather to complement existing diagnostic pathways by increasing clinical awareness based on already available imaging data.

## Data Availability

The datasets used and/or analyzed during the current study are available from the corresponding author on reasonable request.

## Abbreviations

BMD: Bone mineral density
CT: Computed tomography
CBCT: Cone beam computed tomography
CTCI: Computed tomography cortical index
CTI: Computed tomography mandibular index
CTI(I): Computed tomography mandibular index – inferior
CTI(S): Computed tomography mandibular index – superior
CTMI: Computed tomography mental index
DEXA: Dual-energy x-ray absorptiometry
WHO: World Health Organization
FOV: Field of view
HU: Hounsfield Unit
ROI: Region of interest
